# Combined Casein Kinase II inhibition and epigenetic modulation in acute B-lymphoblastic leukemia

**DOI:** 10.1186/s12885-019-5411-0

**Published:** 2019-03-06

**Authors:** Anna Richter, Catrin Roolf, Mohamed Hamed, Yvonne Saara Gladbach, Sina Sender, Christoph Konkolefski, Gudrun Knübel, Anett Sekora, Georg Fuellen, Brigitte Vollmar, Hugo Murua Escobar, Christian Junghanss

**Affiliations:** 1 Department of Medicine, Clinic III – Hematology, Oncology, Palliative Medicine, Rostock University Medical Center, Ernst-Heydemann-Straße 6, 18057 Rostock, Germany; 2 Institute for Biostatistics and Informatics in Medicine and Ageing, Rostock University Medical Center, Ernst-Heydemann-Straße 8, 18057 Rostock, Germany; 3 Faculty of Biosciences, Heidelberg University, Heidelberg, Germany; 4 Division of Applied Bioinformatics, German Cancer Research Center (DKFZ) and National Center for Tumor Diseases (NCT) Heidelberg, Heidelberg, Germany; 5 Small Animal Imaging Core Facility, Rostock University Medical Center, Schillingallee 69a, 18057 Rostock, Germany

**Keywords:** Leukemia, CK2 inhibition, Hypomethylation, In vivo imaging, Methylome analysis

## Abstract

**Background:**

The tumor suppressor protein phosphatase and tensin homolog (PTEN) is a key regulator of the PI3K/AKT pathway which is frequently altered in a variety of tumors including a subset of acute B-lymphoblastic leukemias (B-ALL). While PTEN mutations and deletions are rare in B-ALL, promoter hypermethylation and posttranslational modifications are the main pathways of PTEN inactivation. Casein Kinase II (CK2) is often upregulated in B-ALL and phosphorylates both PTEN and DNA methyltransferase 3A, resulting in increased PI3K/AKT signaling and offering a potential mechanism for further regulation of tumor-related pathways.

**Methods:**

Here, we evaluated the effects of CK2 inhibitor CX-4945 alone and in combination with hypomethylating agent decitabine on B-ALL proliferation and PI3K/AKT pathway activation. We further investigated if CX-4945 intensified decitabine-induced hypomethylation and identified aberrantly methylated biological processes after CK2 inhibition. In vivo tumor cell proliferation in cell line and patient derived xenografts was assessed by longitudinal full body bioluminescence imaging and peripheral blood flow cytometry of NSG mice.

**Results:**

CX-4945 incubation resulted in CK2 inhibition and PI3K pathway downregulation thereby inducing apoptosis and anti-proliferative effects. CX-4945 further affected methylation patterns of tumor-related transcription factors and regulators of cellular metabolism. No overlap with decitabine-affected genes or processes was detected. Decitabine alone revealed only modest anti-proliferative effects on B-ALL cell lines, however, if combined with CX-4945 a synergistic inhibition was observed. In vivo assessment of CX-4945 in B-ALL cell line xenografts resulted in delayed proliferation of B-ALL cells. Combination with DEC further decelerated B-ALL expansion significantly and decreased infiltration in bone marrow and spleen. Effects in patient-derived xenografts all harboring a t(4;11) translocation were heterogeneous.

**Conclusions:**

We herein demonstrate the anti-leukemic potential of CX-4945 in synergy with decitabine in vitro as well as in vivo identifying CK2 as a potentially targetable kinase in B-ALL.

**Electronic supplementary material:**

The online version of this article (10.1186/s12885-019-5411-0) contains supplementary material, which is available to authorized users.

## Background

In B cell acute lymphoblastic leukemia (B-ALL) distinct molecular aberrations contribute to leukemogenesis including mutations, chromosomal translocations or epigenetic dysregulation [1, 2]. The PI3K/AKT pathway induces proliferation, inhibits apoptosis and is involved in B-ALL pathogenesis suggesting potential therapeutic targets [3]. PTEN antagonizes AKT phosphorylation and subsequent pathway activation. As lately reviewed PTEN phosphorylation is considered the most common way of PTEN inactivation in B-ALL in contrast to mutations or deletions in other types of leukemia and solid tumors [4, 5]. Increased phosphorylation of PTEN reduces its phosphatase activity resulting in anti-apoptotic downstream signaling [6].

Apart from increased phosphorylation hypermethylation-induced decreased PTEN transcription has been reported in several tumors including B-ALL [7]. DNA methyltransferase 3A (DNMT3A) overexpression leads to PTEN promoter hypermethylation in chronic eosinophilic leukemia cells [8]. Hypomethylating agents (HMA) can restore the PTEN activity and inhibit PI3K/AKT downstream signaling [8]. In B-ALL inactivation of numerous tumor suppressor genes by aberrant methylation is associated with poor prognosis [9]. Recently we demonstrated that HMA induce apoptosis and cell cycle arrest and inhibit proliferation in human B-ALL [10].

Regulation of DNMT3A activity in B-ALL is not well explored as in contrast to other hematological neoplasms mutations do not seem to occur [11]. Deplus et al. reported that Casein Kinase II (CK2) is able to phosphorylate DNMT3A in osteosarcoma cells and thus mediate genomic methylation [12]. CK2 is a highly conserved serine/threonine kinase frequently overexpressed in solid and hematological neoplasms including B-ALL [13]. CK2 phosphorylates a variety of substrates across numerous pathways and cellular functions. Among those are apoptosis, cell cycle regulation, proliferation and transcription mediated via Wnt, hedgehog, JAK/STAT and NFkB pathways [14]. CK2 further phosphorylates and therefore inactivates tumor suppressor genes like IKZF1 and PTEN in human leukemia cells [15, 16]. In B-ALL cells, Gomes et al. demonstrated that CK2 inhibition by ATP-competitive inhibitor CX-4945 rescued PTEN phosphatase activity accompanied by apoptosis of B-ALL cells [6]. CK2 inhibition also restored IKZF1 tumor suppressor function [15]. Several studies further demonstrated anti-proliferative effects on solid and hematological tumor entities in vitro and in vivo [17–19]. The mechanisms behind CK2’s oncogenic effects and potential therapeutic interventions are currently widely discussed [20].

Here, we hypothesized that CK2 inhibitor CX-4945 alone and in combination with HMA decitabine (DEC) reduces PTEN and AKT phosphorylation and thus inhibits PI3K/AKT mediated proliferation in vitro and in vivo.

## Methods

### Cell lines and cell culture

Human B-ALL precursor cell lines SEM, RS4;11, REH and NALM-6 were purchased from German Collection of Microorganisms and Cell Cultures (DSMZ, Braunschweig, Germany) and cultured as recommended by the manufacturer. Cell lines were selected in order to compare inhibitors in a variety of cell lines of distinct molecular and cytogenetic backgrounds to assess whether inhibitory effects were more prominent in certain subtypes. Media were supplemented with 10% heat-inactivated fetal calf serum (Biochrom, Berlin, Germany) and 100 μg/ml penicillin and streptomycin (Biochrom, Berlin, Germany) and cells were cultured at 37 °C and 5% CO_2_.

### Inhibitory experiments

3.3 × 10^5^ cells per ml were incubated with 5 μM CX-4945 (Hycultec, Beutelsbach, Germany) or 0.1 μM DEC (Selleckchem, Munich, Germany) dissolved in DMSO or respective concentrations of DMSO (controls) for up to 72 h. Apoptosis, proliferation and metabolic activity were assessed as previously described [21]. Experiments were carried out in biological triplicates. Results are described as mean ± standard deviation. Significance (*p* < 0.05) was estimated by 2-tailed student’s t test. Synergy of CX-4945 and DEC combination was calculated by the Bliss independence model [22]. In this test, the observed effect of a combinatory treatment is compared to a calculated expected effect. The expected effect (E) is calculated as follows: E = (A + B) − (A * B), where A and B represent the relative inhibition of single agents A and B. The difference between the observed and expected effect (Δ = O - E) of the combinatory treatment then determines the level of synergy. If the observed inhibitory effect is higher than the expected value (Δ > 0) the combination is referred to as synergistic while Δ < 0 is considered antagonistic. For the calculation, mean values of metabolic activity as well as proliferation after 48 h and 72 h incubation of three independent experiments were used.

### Protein analysis

Cells were lysed using RIPA buffer (Cell Signaling, Danvers, MA, USA) and ultra sound exposure. Proteins were separated on Midi gels (Bio-Rad, Munich, Germany), blotted onto a PVDF membrane (Bio-Rad) using Trans-Blot® Turbo™ Transfer System (Bio-Rad, 2.5 A, 25 V, 10 min), blocked in LI-COR (Lincoln, NE, USA) blocking buffer and detected via LI-COR Odyssey Imaging System and Image Studio Lite software. Antibodies are listed in Additional file 1: Table S1. For quantification, band intensities were assessed using Image Studio Lite software. Values were normalized to respective GAPDH control bands run on each individual gel and ratios between phosphorylated and total protein are given below the images. Mean values and standard deviations of three individual experiments were calculated and displayed as bar charts.

### Genome-wide methylation analysis

After CX-4945 and DEC combined incubation genomic DNA was extracted and bisulfite treated using the NucleoSpin® Tissue Kit (Macherey-Nagel, Dueren, Germany) and peqGOLD Bisulfite Conversion Kit (VWR Peqlab, Erlangen, Germany). The methylation status of the LINE-1 retrotransposon is a marker for global methylation [23]. LINE-1 Methylation specific qPCR (MsqPCR) was carried out in a final volume of 25 μl containing 20 to 50 ng bisulfite treated DNA, Quantitect SYBR Green PCR Master Mix (Qiagen, Hilden, Germany) and primers specific for either methylated or unmethylated DNA as follows: initial denaturation (15 min, 95 °C) followed by 45 cycles of denaturation (15 s, 94 °C), annealing (30 s, 55 °C) and elongation (30 s, 72 °C). Primer sequences are listed in Additional file 2: Table S2.

Whole methylome analyses were carried out at the Genomics & Proteomics Core Facility of the German Cancer Research Center (Heidelberg, Germany) using the Illumina Infinium MethylationEPIC BeadChip platform. Raw and processed data can be retrieved from Gene Expression Omnibus (GSE110454). Raw methylome data was normalized using quantile normalization [24] and summarized to calculate mean beta values [25]. Genes were considered hypomethylated based on a fold change (FC) < 0.25 compared to controls. Gene enrichment analysis was performed using the functional annotation tool DAVID [26] as previously described [27]. Gene ontology (GO) terms annotated to at least two genes and statistically over-represented within the hypomethylated gene set were evaluated by hyper-geometric testing using the entire genome as a universe set and a *p*-value threshold of 0.05. Heatmaps and Venn diagrams were generated using the built-in functions in R [28] and gene-GO term associations were visualized through Chord plots using the GOplot package [29].

### Patients

Mononuclear cells from bone marrow (BM) aspirates of newly diagnosed ALL patients (Rostock University Medical Center) were isolated using Biocoll separating solution (Merck Millipore, Darmstadt, Germany) and previously characterized on a molecular level using next-generation sequencing (Cancer hotspot panel, Ion PGM System, Thermo Fisher Scientific, Schwerte, Germany) according to the manufacturer’s protocol [10]. All experiments were performed in accordance to the Declaration of Helsinki and the local ethical committee standards.

### Animal studies

Stably GFP and enhanced firefly luciferase (ffLuc) transduced SEM and RS4;11 cell lines were kindly provided by Prof. Irmela Jeremias (Helmholtz Center Munich, Germany). SEM and RS4;11 were stably transduced with GFP and enhanced firefly luciferase (ffLuc) in the pCDH-EF1-MCS-T2A-copGFP vector (System Biosciences, Mountain View, CA, USA) using EcoRI and BamHI. Lentivirus production and cell transduction were carried out as described before [30].

NOD scid gamma mice (NOD.Cg-*Prkdc*^*scid*^ *Il2rg*^*tm1Wjl*^/SzJ, NSG, Charles River Laboratories, Sulzfeld, Germany) were bred and housed under specific pathogen-free conditions with access to water and standard chow ad libitum. All experiments were carried out in a laboratory setting and no intervention was performed within the animal housing and breeding rooms. Study group sizes were ten animals for cell line-derived in vivo studies based on biometric Power calculation and at least two animals per group for patient-derived (PDX) experiments [31]. Experiments were approved by the review board of the federal state Mecklenburg-Vorpommern, Germany (reference number: LALLF MV/7221.3–1.1-002/15). Study endpoints for all mice used are listed in Additional file 3 : Table S3 and Additional file 4 : Table S4.

Mice were i.v. injected with 2.5 × 10^6^ cells (SEM-GFP-ffLuc, RS4;11-GFP-ffLuc or de novo B-ALL cells). Tumor cell engraftment was evaluated 7 days after injection via bioluminescence imaging (BLI) using the NightOWL LB 983 in vivo Imaging System (Berthold Technologies, Bad Wildbach, Germany) and Indigo software (Berthold Technologies, version 1.04). For detection animals were intraperitoneally injected with 4.5 mg D-Luciferin (GOLDbiotechnology, St. Louis, MO, USA), anesthetized with ketamine (75 mg/kg) and xylazine (5 mg/kg) and imaged in dorsal and ventral position (60 s exposure, 560 nm emission).

### Therapeutic intervention and evaluation of induced effects

Mice were randomized based on sex, weight (19.1 g – 32.4 g), age (8–16 weeks) and tumor cell engraftment on d7. CX-4945 was dissolved in 0.9% saline containing 5% DMSO. DEC was dissolved in PBS. Animals were simultaneously treated with either vehicle (isotonic saline supplemented with 5% DMSO) or 50 mg/kg i.p. CX-4945 twice daily d7–12 based on previous dose finding studies. For combination studies 0.4 mg/kg DEC was applied i.p. once daily d7-d10. Results were compared to previously published DEC-treated animals [10]. Drug response was analyzed by BLI and peripheral blood (PB) flow cytometry (GFP^+^ or CD19-FITC^+^/CD45-PE^+^ (Becton Dickinson, Heidelberg, Germany)).

At d30 or when PB blast frequency reached 30% (PDX) mice were euthanized by cervical dislocation and leukemic blast frequency was analyzed in PB, BM and spleen. Means and standard deviation of BLI and blast frequency values of all mice of a study group were calculated. Student’s t-test was performed to compare study groups and values < 0.05 were considered significant. May-Grünwald Giemsa staining of PDX spleen cells was carried out as previously described and evaluated by EVOS xl core microscopy [21].

## Results

### CX-4945 affects CK2 activity, PI3K/AKT signaling and cell biology parameters

**Fig. 1:**
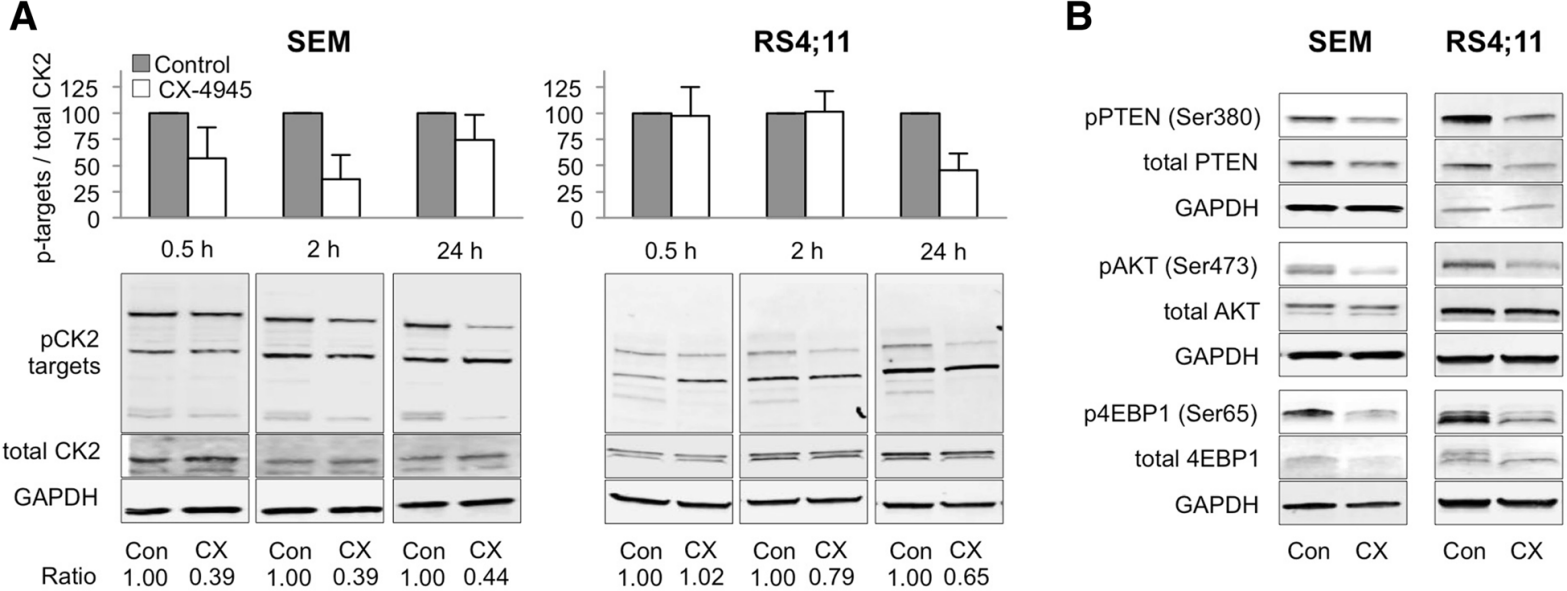
Effects of CX-4945 on CK2 activity and PI3K/AKT pathway phosphorylation in B-ALL cell lines. **a** CK2 activity in SEM and RS4;11 cells was assessed by western blot analysis of phosphorylated CK2 target sites following 2 h (SEM) or 24 h (RS4;11) incubation with CX-4945 (5 μM) (lower panel). For quantification Image Studio Lite software (Version 5.2) was used to determine signal intensities of phosphorylated CK2 target sites and total CK2 alpha 1 protein. Protein bands were normalized to the GAPDH band of the respective sample and the ratio of phosphorylated targets to total CK2A1 protein signal intensities was calculated. Means and SD of three independent experiments are depicted as bar charts in the upper panel. Individual ratios of one representative blot are stated below the respective bands. **b** Activity of PTEN, AKT and 4EBP1 proteins was analyzed by western blot of phosphorylated and total protein forms after 2 h (SEM) or 24 h (RS4;11) incubation with 5 μM CX-4945 (mean + SD; *n* = 3)

SEM and RS4;11 cells were incubated with 5 μM CX-4945 for up to 24 h. Quantification of western blot signals revealed most prominent reductions of phosphorylation of CK2 target sites (pS/pTDXE) [32] after 2 h (SEM) and 24 h (RS4;11) (Fig. 1a). Simultaneous analysis of PTEN, AKT and downstream signaling protein 4EBP1 also showed declined phosphorylation (Fig. 1b).

**Fig. 2:**
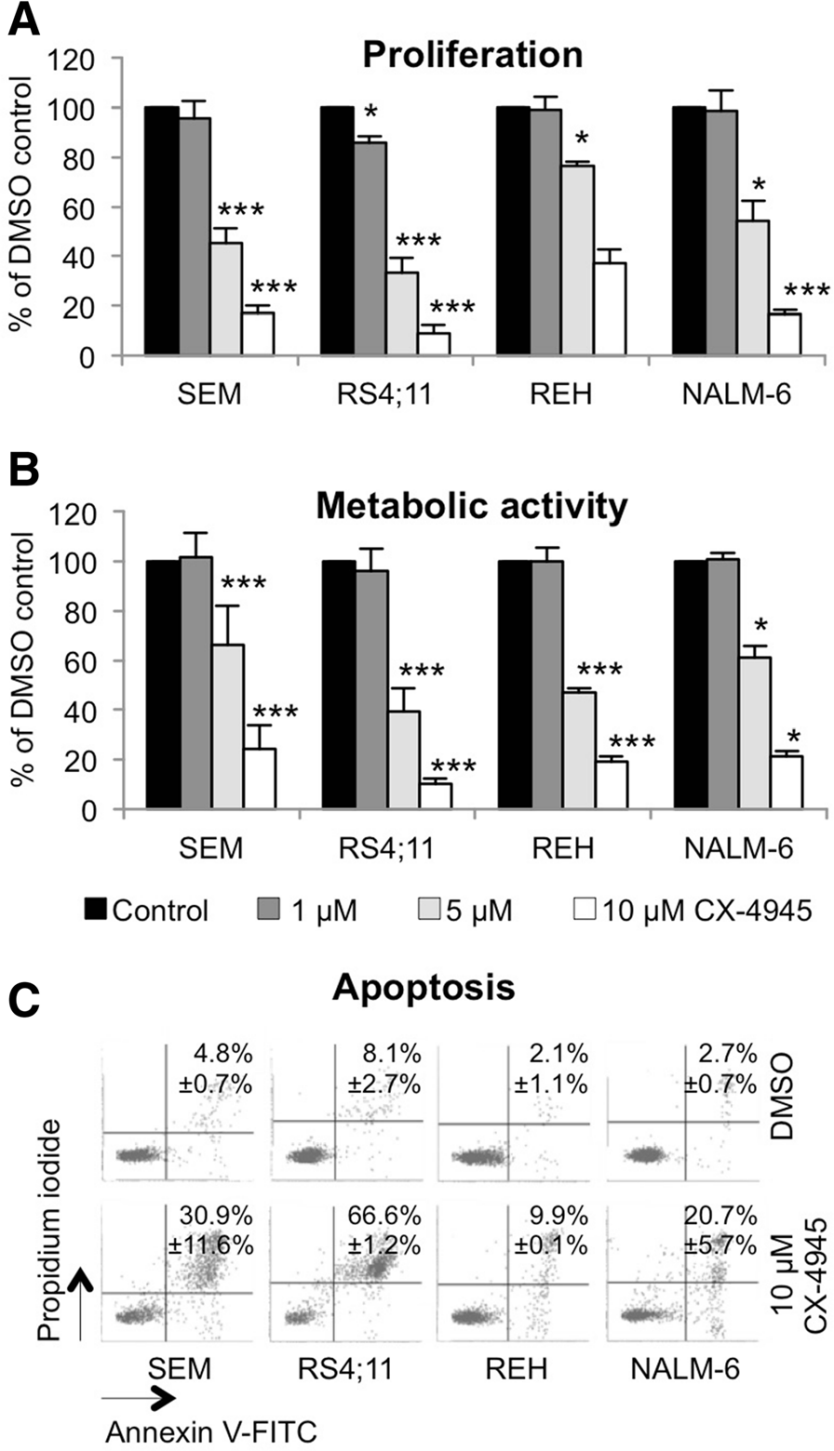
Biological effects of CK2 inhibition on B-ALL cell lines. **a** Proliferation was determined using trypan blue staining after 72 h incubation of increasing CX-4945 concentrations and compared to control cells set to 100% (mean + SD; *n* ≥ 2; * *p* < 0.05, ** *p* < 0.01, *** *p* < 0.005 for *n* ≥ 3 experiments). **b** The WST-1 assay was used to evaluate metabolic activity after 72 h incubation of increasing concentrations of CX-4945. Three technical replicates were performed for each sample (mean + SD; n ≥ 3; * *p* < 0.05, ** p < 0.01, *** *p* < 0.005). **c** The effect on apoptosis was measured by Annexin V-FITC/PI following 72 h CX-4945 incubation. Early apoptotic cells (Annexin V^+^/PI^−^) are located in the lower right quadrant while late apoptotic and necrotic cells (Annexin V^+^/PI^+^) are located in the upper right part. (n ≥ 2)

Compared to controls, proliferation and metabolic activity were significantly decreased in SEM, RS4;11, REH and NALM-6 after 72 h (*p* < 0.05) (Fig. 2a, b). Further, incubation with CX-4945 induced apoptosis with strongest effects in SEM and RS4;11 (Fig. 2c).

### Combined inhibition of CK2 and DNMT3A increases effects synergistically

**Fig. 3:**
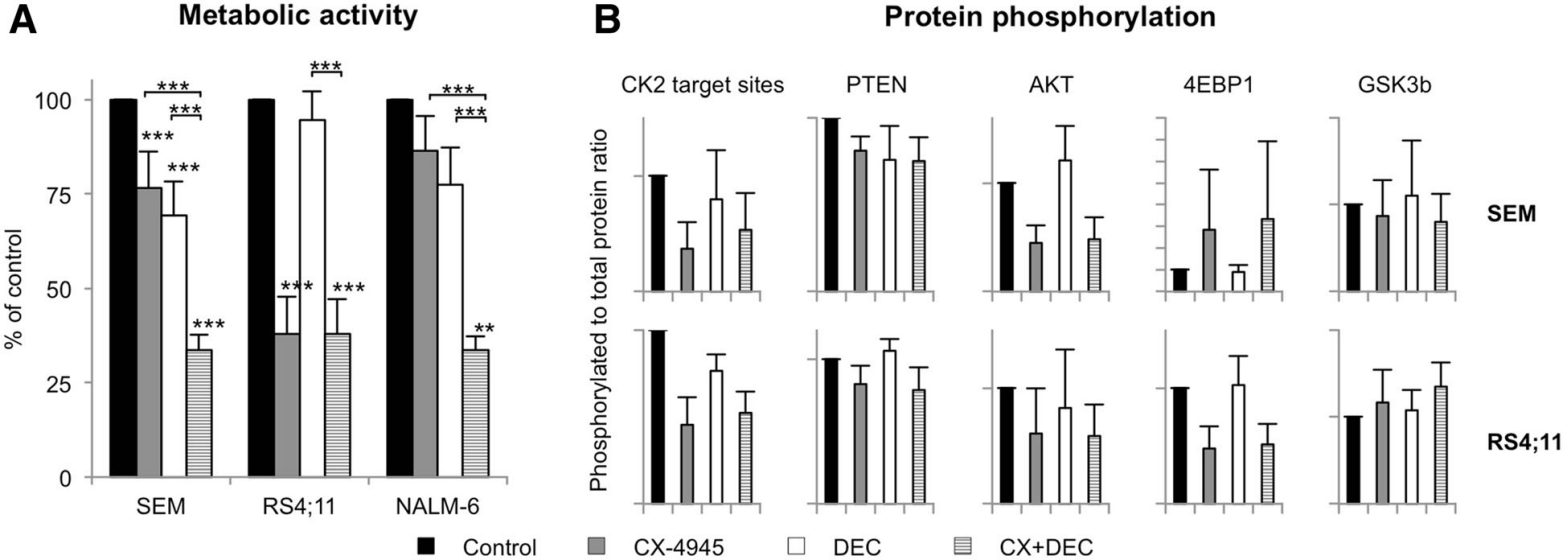
Effects of combined CK2 and DNMT3A inhibition in B-ALL cell lines. **a** The WST-1 metabolism assay was used to evaluate metabolic activity after 72 h incubation of CX-4945 (5 μM) and/or DEC (0.1 μM). Metabolic activity of control cells was set to 100%. Three technical replicates were performed for each sample (mean + SD; n ≥ 3; * *p* < 0.05, ** p < 0.01, *** *p* < 0.005). **b** Phosphorylation of PI3K/AKT pathway proteins was assessed by western blot analysis and subsequent quantification of bands as described before. For protein activity assessment the ratio between phosphorylated and total protein form was calculated and compared to control cells set to 100%. For CK2, phosphorylation of CK2 target sites was determined and compared to the total form of CK2 alpha (mean + SD after 24 h (SEM) or 2 h (RS4;11) incubation with 5 μM CX-4945 and/or 0.1 μM DEC; *n* = 3)

As CK2 is known to influence DNA methylation via DNMT3A phosphorylation CX-4945 was combined with DEC (0.1 μM) for up to 72 h. In DEC-sensitive cell lines SEM and NALM-6 simultaneous application of DEC and CX-4945 significantly reduced the metabolic activity to 33.6 and 33.5%, respectively compared to mono applications (Fig. 3a). The CX-4945 mono application-induced anti-metabolic effect was highest in DEC-resistant RS4;11 cells (37.8%) but addition of DEC did not further decrease metabolic activity. Potential synergistic effects of combined CK2 inhibition and HMA exposure were calculated with Bliss values above 0 indicating synergy. After 72 h, combined application of CX-4945 and DEC demonstrated synergistically reduced metabolic activity in SEM (0.194) and NALM-6 (0.240) but not in RS4;11 (− 0.027) cells. In contrast, no synergistic effect was detected analyzing proliferation. Similar results were obtained after 48 h of incubation (data not shown).

### Synergistic effects are not evoked via PI3K/AKT signaling or intensified hypomethylation

Western blot analyses were performed to evaluate whether addition of DEC to CX-4945 would further influence PI3K/AKT signaling. While DEC alone had hardly any effect on protein phosphorylation combination of DEC and CX-4945 evoked results similar to CX-4945 alone (Fig. 3b). No additional PI3K or MAP kinase pathway regulation was observed (data not shown).

**Fig. 4:**
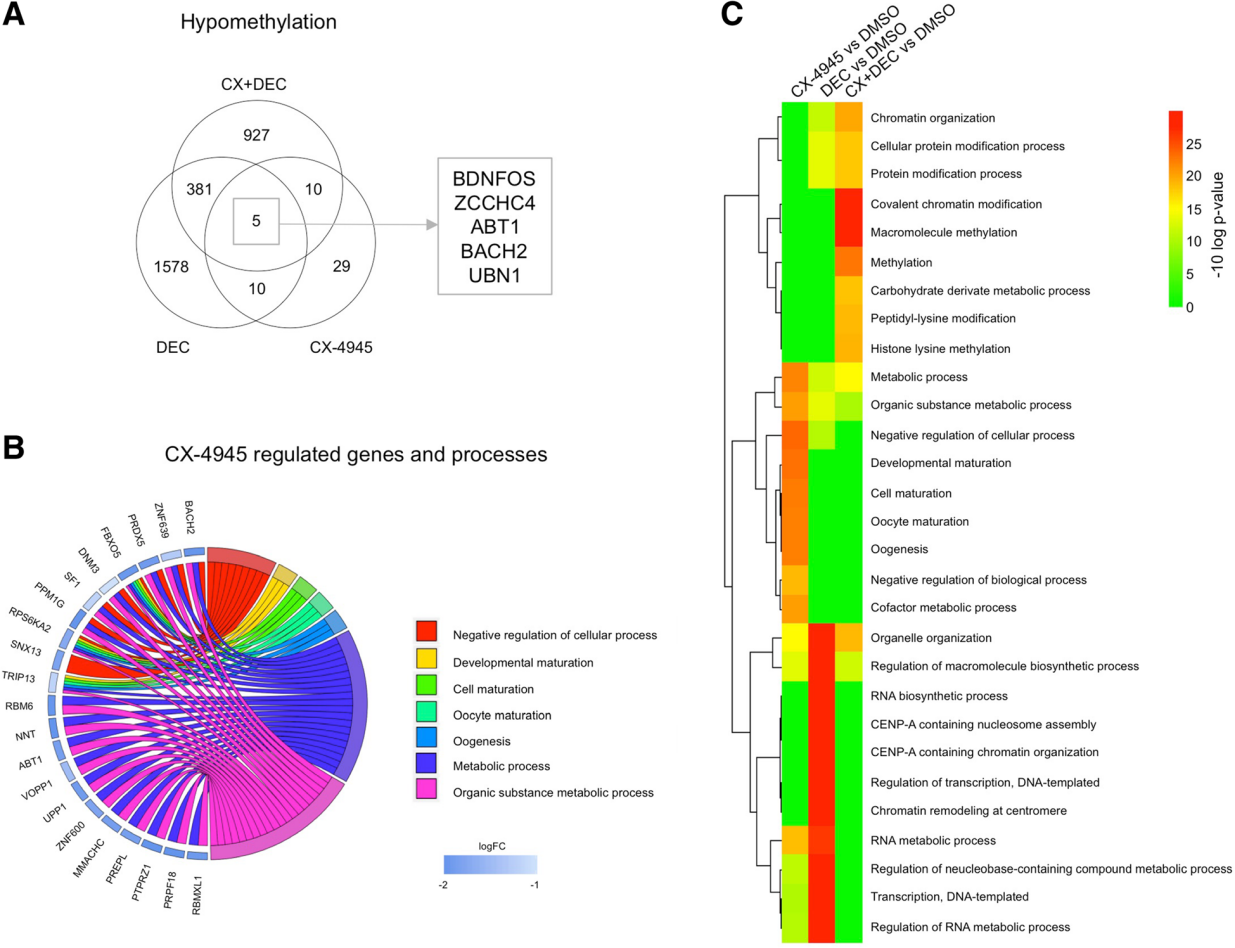
Methylome analysis of SEM cells. **a** The Venn diagram shows the number of hypomethylated genes after 48 h incubation with 5 μM CX-4945, 0.1 μM DEC or both compared to DMSO. Methylation data was normalized using quantile normalization and only changes in methylation of at least 4-fold were considered relevant. **b** Hypomethylated genes were assigned to Gene Ontology (GO) Terms. The chord plot shows the association between the top 7 enriched GO terms and top 30 hypomethylated genes. Genes were further classified by their fold change compared to control cells (blue rectangle). CX-4945 incubation mainly influenced genes annotated by GO terms relating to metabolic processes. FBXO5 and RPS6KA2 have an association to all seven selected GO terms. **c** Average linking hierarchical clustering of the top-10 regulated biological processes of each treatment was performed and visualized in a heatmap based on statistical significance (−10log *p*-values)

Changes in global methylation were assessed by LINE-1 retrotransposon MsqPCR and revealed no hypomethylating effect of CX-4945 (Additional file 5: Figure S1). To evaluate whether the observed synergistic anti-proliferative effect was evoked by potential gene-specific hypomethylation of tumor suppressor genes or signaling proteins genome-wide methylome analysis was performed. Compared to controls DEC reduced methylation of 1578 genes while the combination of both drugs induced hypomethylation in 927 genes (FC < 0.25). After CX-4945 incubation 54 genes were hypomethylated (Fig. 4a, Additional file 6: Table S5 and Additional file 7: Tables S6). Methylation of only 5 genes (BDNFOS, ZCCHC4, ABT1, BACH2, UBN1) was reduced by both mono applications as well as combined incubation. Among these were transcription factors ABT1 (activator of basal transcription 1) and BACH2 (basic leucine zipper transcription factor). CX-4945-hypomethylated genes mainly affected cellular process regulation, maturation and metabolism (Fig. 4b, c; Additional file 8: Table S7). The GO terms associated to genes regulated by combined CK2 inhibition and DEC exposure were chromatin modification and methylation (Additional file 9: Figure S2, Additional file 10: Table S8). Interestingly, there was marginal overlap between CX-4945, DEC or combined therapy affected processes (Fig. 4c) and none of the drugs changed the promoter methylation pattern of PTEN or CK2 (Additional file 11: Table S9).

### Inhibition of CK2 alone and in combination with DEC delays tumor cell proliferation in vivo

**Fig. 5:**
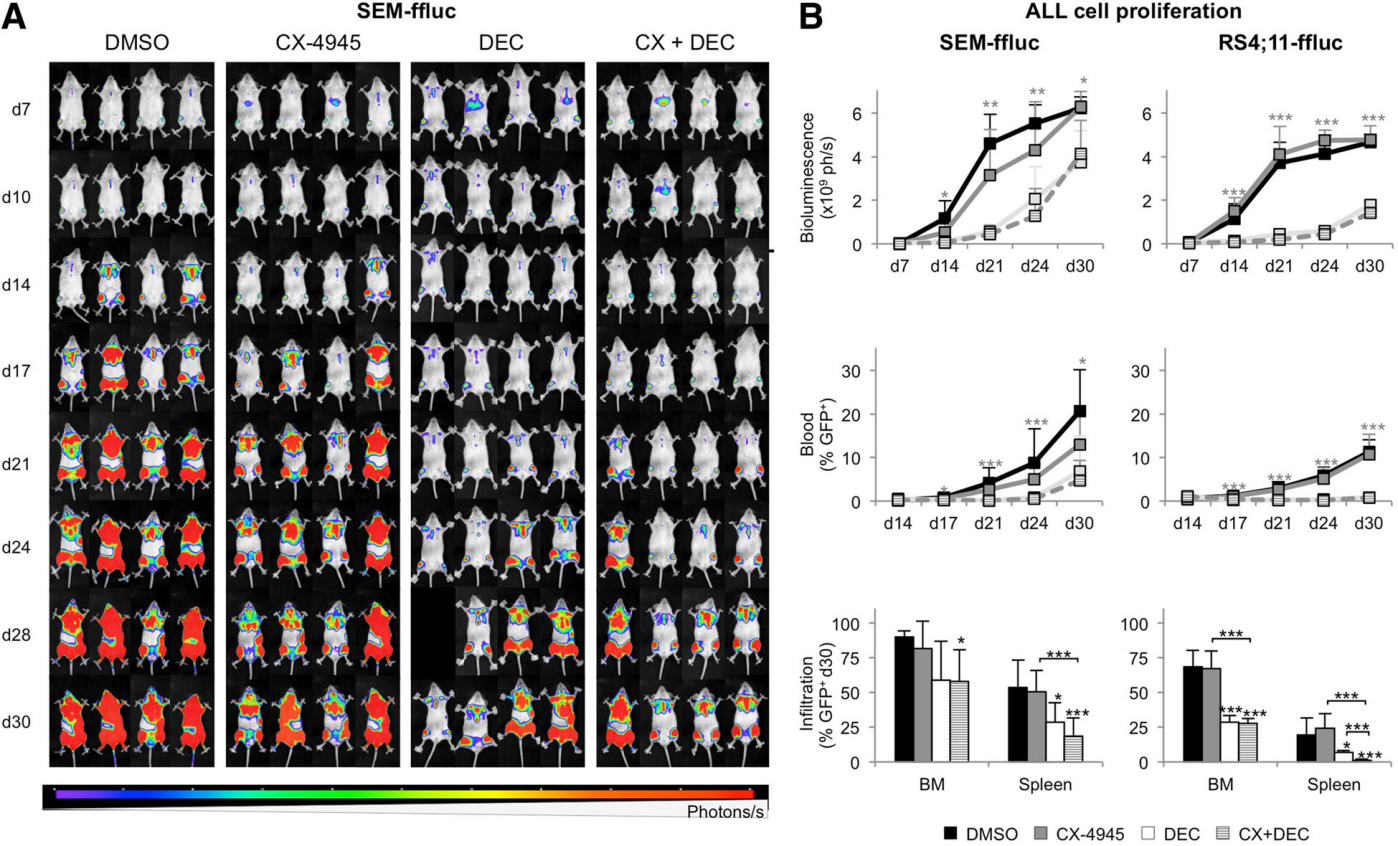
In vivo evaluation of CX-4945 and DEC. **a** Longitudinal observation of leukemic blast proliferation in four representative SEM-ffluc xenograft mice is displayed following vehicle (NaCl supplemented with 5% DMSO, BID d7–12), CX-4945 (50 mg/kg body weight (BW), BID d7–12), DEC (0.4 mg/kg BW, d7–10) or CX-4945 plus DEC treatment over a period of 30 days. Increasing luminescence (ph/s) is proportional to proliferation of luciferase-expressing SEM-ffluc cells. **b** Quantification of full body bioluminescence (ph/s) after treatment in SEM-ffluc and RS4;11-ffluc animals was performed by adding total luminescence signals of dorsal and ventral imaging. Significant differences are indicated for combined treatment of CX-4945 and DEC vs CX-4945 mono application. CX-4945 alone induced no significant change in full body bioluminescence compared to controls (mean + SD; * *p* < 0.05, ** *p* < 0.01, *** *p* < 0.005). Blast frequency in PB was assessed by flow cytometry measurement of GFP^+^ SEM-ffluc and GFP^+^ RS4;11-ffluc cells over a period of 30 days. Significant differences are indicated for combined therapy vs CX-4945 mono application (mean + SD; * *p* < 0.05, *** *p* < 0.005). Infiltration of leukemic blasts was analyzed in BM and spleen after euthanasia on day 30. The amount of GFP^+^ SEM-ffluc and RS4;11-ffluc blasts was determined by flow cytometry (mean + SD; * *p* < 0.05, *** *p* < 0.005)

The anti-leukemic activity of CX-4945 was investigated in vivo using orthotopic SEM-GFP-ffLuc- and RS4;11-GFP-ffLuc-derivded xenograft mouse models. These t(4;11)-harbouring cell lines were selected based on most promising results in vitro. Only healthy, age-, weight-, sex- and engraftment-matched animals were included in study groups. Therapeutic response was analyzed using longitudinal full body BLI and flow cytometric detection of GFP expression in PB, spleen and BM (Fig. 5). BLI signals directly correlate to the number of luciferase^+^ tumor blasts and are therefore used as a direct marker for tumor cell proliferation.

In SEM-derived xenografts CX-4945 delayed B-ALL cell proliferation until day 24 (CX-4945 4.3 ± 2.2 × 10^9^ ph/s; control 5.5 ± 0.8 × 10^9^ ph/s) before reaching BLI signal levels similar to the controls on day 30. Animals also showed reduced PB blast counts throughout the entire examination period (d30, CX-4945 12.9 ± 7.2%; control 20.6 ± 9.5%). DEC alone induced stronger effects than CX-4945 mono therapy with significantly decreased BLI signals (d24, 2.1 ± 1.5 × 10^9^ ph/s) as well as PB, spleen and BM blast frequencies (Fig. 5b). Combined CX-4945 and DEC therapy was more effective than DEC alone and significantly decreased bioluminescence signals and blast frequency compared to CX-4945 mono application (d24, 1.3 ± 1.3 × 10^9^ ph/s). Further, leukemic infiltration of BM and spleen was significantly reduced compared to controls.

CX-4945 had no influence on RS4;11-derived xenografts (Fig. 5b). Addition of DEC resulted in significantly reduced B-ALL cell proliferation indicated by delayed BLI signal increase (d30, control 4.7 ± 0.4 × 10^9^ ph/s; CX-4945 4.8 ± 0.6 × 10^9^ ph/s; DEC 1.8 ± 0.2 × 10^9^ ph/s; CX + DEC 1.4 ± 0.5 × 10^9^ ph/s) as well as PB, BM and spleen blast counts.

### CX-4945 in combination with DEC influences patient-derived B-ALL cell proliferation in vivo

To increase comparability of animal studies and human leukemia patients, xenografts of three B-ALL patients were established in NSG mice and treated with CX-4945, DEC or in combination until control animals reached a PB blast amount of 30%. To ensure comparability between cell line- and patient-derived xenografts, only MLL-rearranged samples were engrafted.

**Fig. 6:**
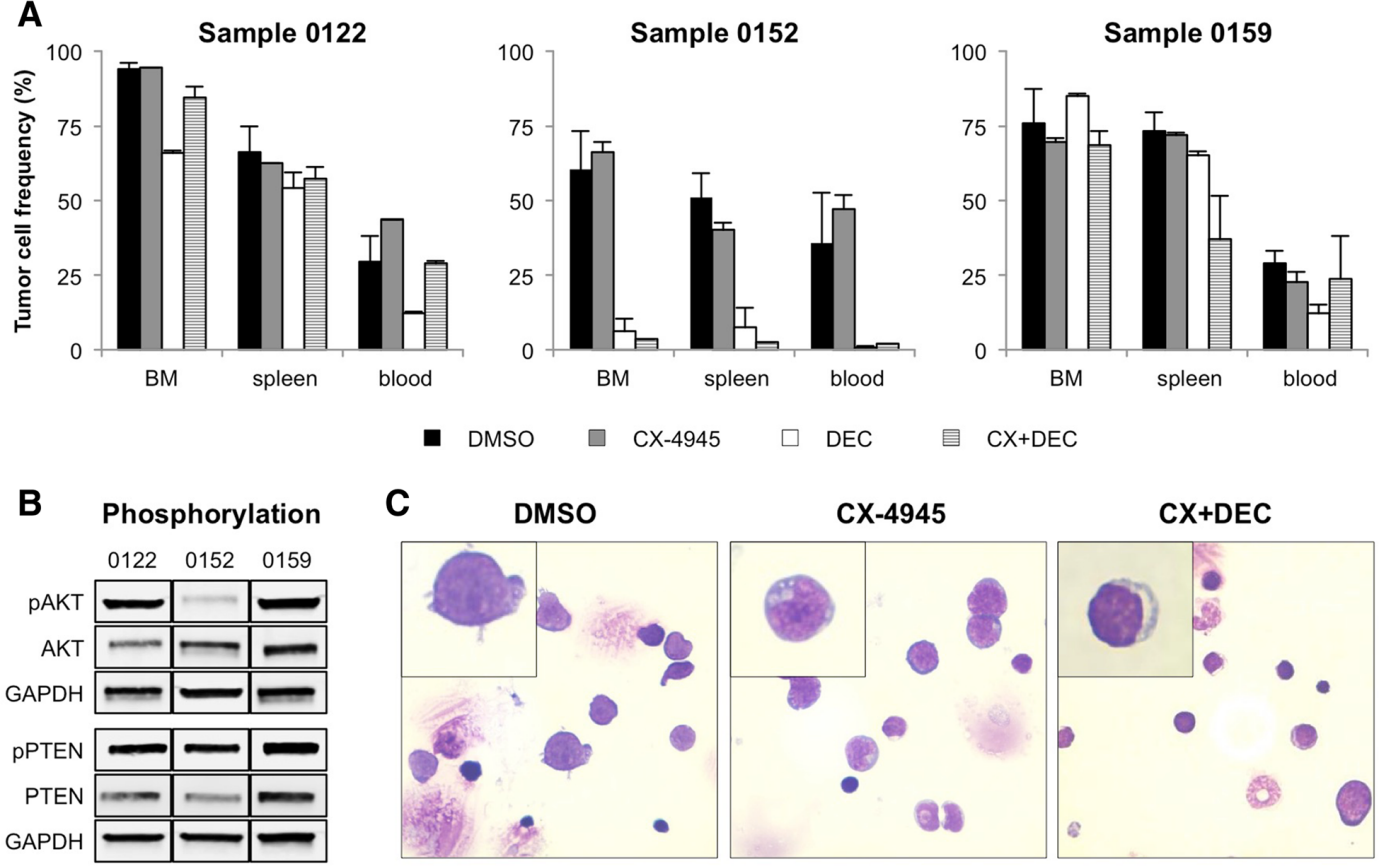
Evaluation of CX-4945 and DEC in patient-derived xenograft mice. **a** Xenograft mice of three different B-ALL patients (0122, 0152, 0159; patient characteristics were reported previously) were treated with vehicle (NaCl supplemented with 5% DMSO; BID d7–12), CX-4945 (50 mg/kg BW, BID, d7–12) or CX-4945 plus DEC (0.4 mg/kg BW, d7–10) and euthanized when PB blast frequency in controls reached 25%. The amount of patient-derived B-ALL tumor cells in BM, spleen and PB was assessed by CD45-FITC/CD19-PE staining and subsequent flow cytometry (mean + SD; vehicle: *n* = 4–5 animals per patient, treatment groups: *n* = 1–2 animals per patient). **b** AKT and PTEN activity status was analyzed in PDX mice spleen cells by western blot of phosphorylated and total protein forms. **c** Cytospin preparations of cells isolated from spleens of patient 0152 derived mice. Slides were stained with Pappenheim and evaluated using the EVOS xl core microscope and 100x magnification)

Treatment with CX-4945 did not decrease leukemic blasts in BM, spleen or PB in PDX mice (Fig. 6a). Combination with DEC as well as DEC incubation alone resulted in a strong reduction of tumor load in patient 0152-derived mice. Interestingly, basal AKT activity in spleen cells was reduced in patient 0152 xenografts compared to other PDX samples (Fig. 6b). Phosphorylation of PTEN and other PI3K pathway molecules was similar in all samples (data not shown). Cytospin preparations of spleen cells revealed no changes in blast morphology after CX-4945 or combined therapy (Fig. 6c, inset). However, PB blast frequency was decreased combinatory treatment (control 36.0%; CX + DEC 2.0%) (Fig. 6a).

In patient 0159-derived mice combined application of CX-4945 and DEC lowered spleen infiltration while the tumor burden in BM and PB remained constant. Only minor changes in leukemic blast frequency were observed in animals bearing patient 0122-derived tumors.

## Discussion

In addition to mutations and structural changes of DNA several mechanisms and pathways are involved in B-ALL pathogenesis including posttranslational modifications and aberrant methylation. Thus combined targeting of tumor-initiating pathways is advantageous and currently evaluated in a variety of approaches in preclinical and clinical settings. Although CK2 inhibition and hypomethylation of tumor suppressor gene promoters show promising results in hematological neoplasms the effect of their combined application has not yet been addressed. Simultaneous CK2 inactivation and hypomethylation may shed light on potential crosstalk between both mechanisms and potential synergistic anti-proliferative effects on B-ALL cells.

In B-ALL increased promoter hypermethylation is a typical feature observed in relapse, often silencing tumor suppressor genes such as PTEN [5, 33]. PTEN is further inhibited by CK2-mediated phosphorylation. CK2 is frequently upregulated in hematological neoplasms and a key regulator of diverse cellular mechanisms including cell cycle, apoptosis and transcription [14, 34, 35].

In our study we found that CX-4945 inhibits CK2 resulting in decreased phosphorylation of PI3K pathway signaling proteins and subsequent reduction of B-ALL cell proliferation and metabolic activity as well as increased apoptosis rates. Other studies of CK2 inhibition on B-ALL cell lines also found PI3K pathway downregulation and reduced cell viability accompanied by apoptosis induction [6, 15, 36, 37]. Methylome analysis also revealed a strong influence of CX-4945 on the methylation of metabolism-related genes, matching the data obtained by WST-1 metabolic activity assay. Results within an in vivo human B-ALL xenograft model matched in vitro anti-proliferative results. Bioluminescence imaging also revealed delayed leukemic proliferation and reduced blast frequency in SEM-derived mice. Whether these effects were due to cytotoxicity or due to cell death induction has to be determined in further experiments. Matching our in vitro data Song et al. demonstrated reduced leukemic progression and an improved overall survival in a NALM-6-derived xenograft model after 10 days of treatment using higher concentrations than in our study [15]. Other groups also discovered anti-proliferative potential of CK2 inhibition in CLL [38, 39] and T-ALL in vivo studies [40].

We then added DEC to CX-4945 treatment in order to identify potential enhanced pathway and methylome modulation. We found that combined application of CX-4945 and DEC acted synergistically in vitro but while PI3K pathway phosphorylation mirrors CX-4945 mono treatment, methylome patterns of combined application show no overlap with either single application. This suggests that a previously unconsidered mechanism is responsible for the observed synergistic anti-proliferative effects. We hypothesized that hypomethylation and thus activation of PTEN might be responsible for decreased proliferation and metabolic activity but could not detect significantly lower methylation or phosphorylation after combined therapy. However, the transcription factor BACH2 is a potential linker between CX-4945 and DEC-induced effects. We found that BACH2 is hypomethylated by CX-4945, DEC and combined treatment while Ando et al. demonstrated that it can be phosphorylated by PI3K pathway proteins [41]. Regulating B cell development at the pre-B cell receptor checkpoint BACH2 functions as a tumor suppressor in B-ALL [42–44]. Further, Ge et al. recently demonstrated that both BACH2 and its counterpart BCL6 interact with CK2 target Ikaros (IKZF1) resulting in increased BACH2 expression and reduced B-ALL proliferation [45]. Epigenetic modulation of the BACH2/BCL6 axis may therefore be an additional mechanism to regulate tumor cell apoptosis and proliferation. However, the importance of CX-4945-mediated loss of CK2 kinase activity for the observed effects is not yet clear as DEC mono therapy was not significantly inferior to combined CX-4945 and DEC treatment during the observation period in vivo. Prolonged therapeutic intervention or higher CX-4945 doses might be beneficial for the treatment outcome as stated above. It further seems possible that, although no significant synergistic anti-proliferative effect was observed, CX-4945 alone or in combination with DEC influences biochemical, metabolic or other signaling pathways in tumor blasts.

As the PI3K/AKT signaling pathway can be aberrantly activated in B-ALL patients we further tested CX-4945 in three B-ALL patient-derived xenograft models all harboring a t(4;11) translocation [6. Independently of patients’ PTEN and AKT phosphorylation status or hotspot mutations no satisfactory anti-leukemic effect was achieved. This matches the findings of Prins et al. who incubated 56 primary pediatric and adult B-ALL samples with increasing concentrations of CX-4945 and found reduced tumor cell viability in only 3 (5.2%) samples compared to much higher response rates in T-ALL, CLL and lymphoma [38, 46]. In contrast Song et al. found a reduced number of blasts following CX-4945 therapy in high risk B-ALL xenografts. [15] This discrepancy might be explained by the fact that only patients with a t(4;11) were examined or by different application schemes. While Song et al. treated mice with 100 mg/kg daily for 22 days and analyzed organs directly after the last application we used a shorter application regime (50 mg/kg twice daily for 6 days) and evaluated the blast frequency after 30 days or more. It thus appears that CX-4945 delays leukemic blast proliferation during therapy while this effect diminishes when the drug is removed. This hypothesis also matches our findings from cell line-derived B-ALL xenograft observations. Evaluation of pharmacokinetic parameters demonstrated long half-life of CX-4945 in rats (14.7 h and 10.9 h for intravenous and oral administration, respectively) [47] while Siddiqui-Jain et al. found high clearance rates accompanied by rapidly decreasing plasma concentrations in mice [48]. Further pharmacokinetic evaluation is therefore necessary. Alternative application schemes might be beneficial and should be addressed in future studies.

## Conclusion

Taken together we herein demonstrate that the novel strategy of combined CK2 inhibition and DEC application significantly decreases B-ALL proliferation. In this study we evaluated PI3K/AKT signaling, methylome patterns and therapeutic response in vivo after single and combined application. Although CK2 is known to interact with both, PI3K/AKT and DNMT3A-mediated methylation neither pathway can be made fully responsible for the observed synergistic effects [12]. Therefore, further experiments need to be conducted to identify the underlying mechanism of the demonstrated synergistic anti-tumor potential.

## Additional files

Additional file 1: **Table S1.** List of antibodies used for Western blot analyses. (DOCX 14 kb)

Additional file 2: **Table S2.** List of primers used for LINE-1 methylation analysis. (DOCX 13 kb)

Additional file 3: **Table S3.** List of cell line derived xenograft mice used (DOCX 16 kb)

Additional file 4: **Table S4.** List of patient derived xenograft mice used (DOCX 16 kb)

Additional file 5: **Figure S1.** Evaluation of CX-4945- and DEC-induced effects on global methylation in SEM and RS4;11 cells. Global methylation was analyzed using methylation specific qPCR of the LINE-1 retrotransposon after 48 h incubation of CX-4945 (5 μM) and/or DEC (0.1 μM). LINE-1 methylation of control cells was set to 100 % (three technical replicates per sample; mean + SD; n=3; *p<0.05, **p<0.01, ***p<0.005). (JPG 16 kb)

Additional file 6: **Table S5.** List of genes that were hypomethylated after CX-4945 incubation (FC<0.25) compared to controls. (XLSX 11 kb)

Additional file 7: **Table S6.** List of genes that were hypomethylated after combined CX-4945 and DEC incubation (FC<0.25) compared to controls. (XLSX 48 kb)

Additional file 8: **Table S7.** List of Biological processes regulated by CX-4945 incubation. (XLSX 15 kb)

Additional file 9: **Figure S2.** Genes and processes highly regulated by combined CX-4945 and DEC incubation. Hypomethylated genes were assigned to Gene Ontology (GO) Terms. The chord plot shows the association between the top 7 enriched GO terms and top 30 hypomethylated genes. Genes are further classified by their fold change compared to control cells (blue rectangle). (JPG 41 kb)

Additional File 10: **Table S8.** List of Biological processes regulated by combined CX-4945 and DEC incubation. (XLSX 13 kb)

Additional File 11: **Table S9.** Influence of CX and DEC on PTEN and CK2 promoter methylation beta values and respective fold changes (FC). (DOCX 14 kb)

## Abbreviations

ALL: Acute lymphoblastic leukemia
BLI: Bioluminescence imaging
BM: Bone marrow
CK2: Casein Kinase II
DEC: Decitabine
FC: Fold change
ffluc: Firefly luciferase
GFP: Green fluorescent protein
GO: Gene ontology
HMA: Hypomethylating agent
MsqPCR: Methylation specific qPCR
NSG: NOD scid gamma
PB: Peripheral blood
PDX: Patient-derived xenograft
