# Supplementary material for: Combined Casein Kinase II inhibition and epigenetic modulation in acute B-lymphoblastic leukemia
Source: BMC Cancer. 2019 Mar 6;19:202. doi: 10.1186/s12885-019-5411-0 (PMC6404304; doi:10.1186/s12885-019-5411-0)
Supplement: Supplementary file 1 — Table S1. List of antibodies used for Western blot analyses. (DOCX 14 kb) [file 12885_2019_5411_MOESM1_ESM.docx]

Supplemental Table 1: List of antibodies used for Western blot analyses

| Antibody | Host | Clone | Dilution | Supplier |
| --- | --- | --- | --- | --- |
| GAPDH | Mouse | ZG003 | 1:20,000 | Invitrogen (Carlsbad, CA, USA) |
| Phospho CK2 substrates | Rabbit | Monoclonal mix | 1:1,000 | Cell Signaling (Danvers, MA, USA) |
| CK2A1 | Rabbit | polyclonal | 1:1,000 | Cell Signaling (Danvers, MA, USA) |
| Phospho PTEN (Ser380) | Rabbit | polyclonal | 1:1,000 | Cell Signaling (Danvers, MA, USA) |
| PTEN | Rabbit | 138G6 | 1:1,000 | Cell Signaling (Danvers, MA, USA) |
| Phospho AKT (Ser473) | Rabbit | polyclonal | 1:500 | Cell Signaling (Danvers, MA, USA) |
| AKT | Rabbit | polyclonal | 1:1,000 | Cell Signaling (Danvers, MA, USA) |
| Phospho 4EBP1 (Ser65) | Rabbit | 174A9 | 1:1,000 | Cell Signaling (Danvers, MA, USA) |
| 4EBP1 | Rabbit | polyclonal | 1:1,000 | Cell Signaling (Danvers, MA, USA) |
| Phospho GSK3B (Ser9) | Rabbit | 5B3 | 1:500 | Cell Signaling (Danvers, MA, USA) |
| GSK3B | Rabbit | 27C10 | 1:1,000 | Cell Signaling (Danvers, MA, USA) |
| IRDye 680 goat anti-mouse | Goat |  | 1:20,000 | LI-COR (Lincoln, NE, USA) |
| IRDye 800 goat anti-mouse | Goat |  | 1:20,000 | LI-COR (Lincoln, NE, USA) |
| IRDye 680 goat anti-rabbit | Goat |  | 1:20,000 | LI-COR (Lincoln, NE, USA) |
| IRDye 800 goat anti-rabbit | Goat |  | 1:20,000 | LI-COR (Lincoln, NE, USA) |
