# Supplementary material for: Combined Casein Kinase II inhibition and epigenetic modulation in acute B-lymphoblastic leukemia
Source: BMC Cancer. 2019 Mar 6;19:202. doi: 10.1186/s12885-019-5411-0 (PMC6404304; doi:10.1186/s12885-019-5411-0)
Supplement: Supplementary file 2 — Table S2. List of primers used for LINE-1 methylation analysis. (DOCX 13 kb) [file 12885_2019_5411_MOESM2_ESM.docx]

Supplemental Table 1: List of primers used for LINE-1 methylation analysis.

| Primer | Sequence |
| --- | --- |
| LINE-1_forw_unmethylated | 5‘ TGTGTGTGAGTTGAAGTAGGGT 3’ |
| LINE-1_rev_unmethylated | 5’ ACCCAATTTTCCAAATACAACCATCA 3’ |
| LINE-1_forw_methylated | 5’ CGCGAGTCGAAGTAGGGC 3’ |
| LINE-1_rev_methylated | 5’ ACCCGATTTTCCAAATACGACCG 3’ |
