# Supplementary material for: Combined Casein Kinase II inhibition and epigenetic modulation in acute B-lymphoblastic leukemia
Source: BMC Cancer. 2019 Mar 6;19:202. doi: 10.1186/s12885-019-5411-0 (PMC6404304; doi:10.1186/s12885-019-5411-0)
Supplement: Supplementary file 3 — Table S3. List of cell line derived xenograft mice used (DOCX 16 kb) [file 12885_2019_5411_MOESM3_ESM.docx]

Supplemental Table 2: List of cell line derived xenograft mice used

|  | SEM-ffluc | | RS4;11-ffluc | |
| --- | --- | --- | --- | --- |
| Treatment | Mouse ID | Study endpoint (days) | Mouse ID | Study endpoint (days) |
| Saline +  5% DMSO | NSG-101 | 30 | NSG-130 | 30 |
|  | NSG-127 | 30 | NSG-137 | 31 |
|  | NSG-128 | 31 | NSG-139 | 31 |
|  | NSG-129 | 31 | NSG-146 | 31 |
|  | NSG-150 | 30 | NSG-147 | 30 |
|  | NSG-151 | 30 | NSG-148 | Unexpected †d21 |
|  | NSG-152 | 30 | NSG-155 | 30 |
|  | NSG-163 | 29 | NSG-156 | 31 |
|  | NSG-165 | 29 | NSG-157 | 31 |
|  | NSG-166 | 29 | NSG-169 | 29 |
|  | NSG-168 | 30 | NSG-171 | 30 |
|  | NSG-175 | 30 | NSG-173 | 30 |
| CX-4945 | NSG-81 | 30 | NSG-111 | 30 |
|  | NSG-83 | 30 | NSG-115 | 30 |
|  | NSG-86 | 30 | NSG-120 | 30 |
|  | NSG-88 | 30 | NSG-121 | 31 |
|  | NSG-92 | 30 | NSG-122 | 30 |
|  | NSG-94 | 30 | NSG-123 | 31 |
|  | NSG-149 | 30 | NSG-158 | 31 |
|  | NSG-153 | 30 | NSG-159 | 31 |
|  | NSG-162 | 29 | NSG-160 | 31 |
|  | NSG-174 | 30 | NSG-170 | 29 |
|  |  |  | NSG-172 | 30 |
| DEC | NSG-31 | Anesthesia †d14 | NSG-104 | Anesthesia †d22 |
|  | NSG-32 | 30 | NSG-105 | Anesthesia †d22 |
|  | NSG-35 | 30 | NSG-112 | 30 |
|  | NSG-38 | 30 | NSG-113 | 30 |
|  | NSG-44 | Unexpected †d16 | NSG-118 | 30 |
|  | NSG-52 | 31 | NSG-119 | 31 |
|  | NSG-53 | 30 | NSG-125 | 30 |
|  | NSG-59 | 31 | NSG-126 | 31 |
|  | NSG-60 | 31 | NSG-141 | 30 |
| CX+DEC | NSG-89 | 30 | NSG-131 | 30 |
|  | NSG-93 | 30 | NSG-132 | 31 |
|  | NSG-96 | 30 | NSG-133 | 30 |
|  | NSG-97 | 30 | NSG-134 | 31 |
|  | NSG-98 | Unexpected †d12 | NSG-142 | 30 |
|  | NSG-102 | 30 | NSG-143 | 30 |
|  | NSG-103 | Anesthesia †d22 | NSG-144 | Unexpected †d21 |
|  |  |  | NSG-145 | 30 |
