# Supplementary material for: Combined Casein Kinase II inhibition and epigenetic modulation in acute B-lymphoblastic leukemia
Source: BMC Cancer. 2019 Mar 6;19:202. doi: 10.1186/s12885-019-5411-0 (PMC6404304; doi:10.1186/s12885-019-5411-0)
Supplement: Supplementary file 4 — Table S4. List of patient derived xenograft mice used (DOCX 16 kb) [file 12885_2019_5411_MOESM4_ESM.docx]

Supplemental Table 3: List of patient derived xenograft mice used

|  | Sample 0122 | | | Sample 0152 | | | Sample 0159 | | |
| --- | --- | --- | --- | --- | --- | --- | --- | --- | --- |
| Treatment | Mouse ID | BLI | Study endpoint (days) | Mouse ID | BLI | Study endpoint (days) | Mouse ID | BLI | Study endpoint (days) |
| Saline | PDX-29 | - | 29 | PDX-33 | - | 53 | PDX-24 | - | Unexpected †d25 |
|  | PDX-50 | X | 35 | PDX-35 | - | 53 | PDX-26 | - | 31 |
|  | PDX-51 | X | 35 | PDX-56 | - | 43 | PDX-45 | X | 36 |
|  | PDX-59 | X | 35 | PDX-62 | - | 43 | PDX-46 | X | 36 |
|  | PDX-65 | X | 35 | PDX-71 | - | 46 | PDX-68 | X | 36 |
| CX-4945 | PDX-49 | X | 35 | PDX-57 | - | 43 | PDX-45 | X | 36 |
|  | PDX-53 | X | Anesthesia †d14 | PDX-69 | - | 46 | PDX-46 | X | 36 |
| DEC | PDX-30 | - | 29 | PDX-34 | - | 53 | PDX-25 | - | 31 |
|  | PDX-31 | - | 29 | PDX-36 | - | 53 | PDX-27 | - | 31 |
| CX+DEC | PDX-58 | X | 35 | PDX-64 | - | 43 | PDX-60 | X | 36 |
|  | PDX-66 | X | 35 |  |  |  | PDX-67 | X | 36 |

BLI, bioluminescence imaging
