# Supplementary figures and images for: Combined Casein Kinase II inhibition and epigenetic modulation in acute B-lymphoblastic leukemia
Source: BMC Cancer. 2019 Mar 6;19:202. doi: 10.1186/s12885-019-5411-0 (PMC6404304; doi:10.1186/s12885-019-5411-0)

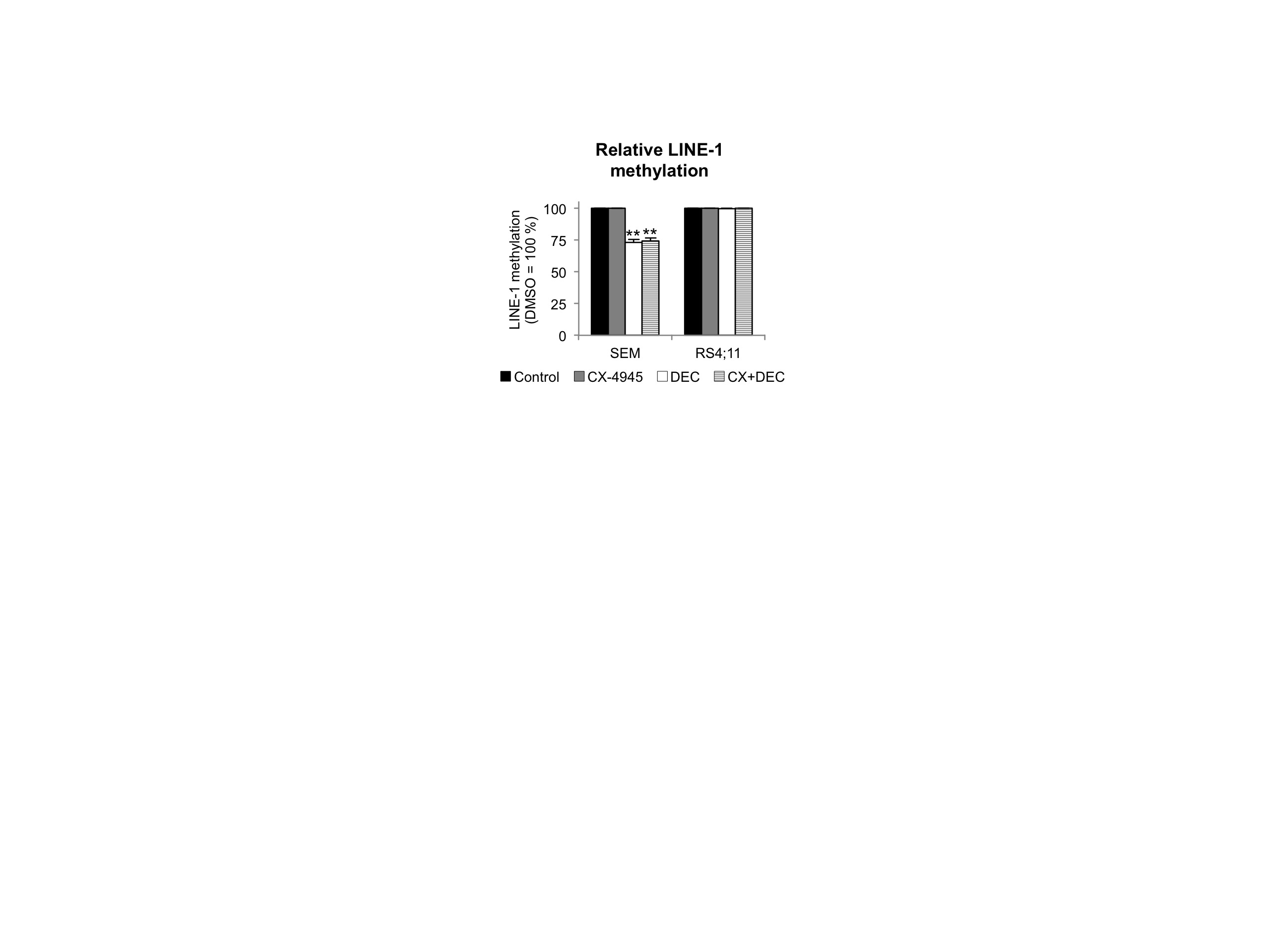

Supplement: Supplementary file 5 — Figure S1. Evaluation of CX-4945- and DEC-induced effects on global methylation in SEM and RS4;11 cells. Global methylation was analyzed using methylation specific qPCR of the LINE-1 retrotransposon after 48 h incubation of CX-4945 (5 μM) and/or DEC (0.1 μM). LINE-1 methylation of control cells was set to 100 % (three technical replicates per sample; mean + SD; n=3; *p<0.05, **p<0.01, ***p<0.005). (JPG 16 kb) [file 12885_2019_5411_MOESM5_ESM.jpg]

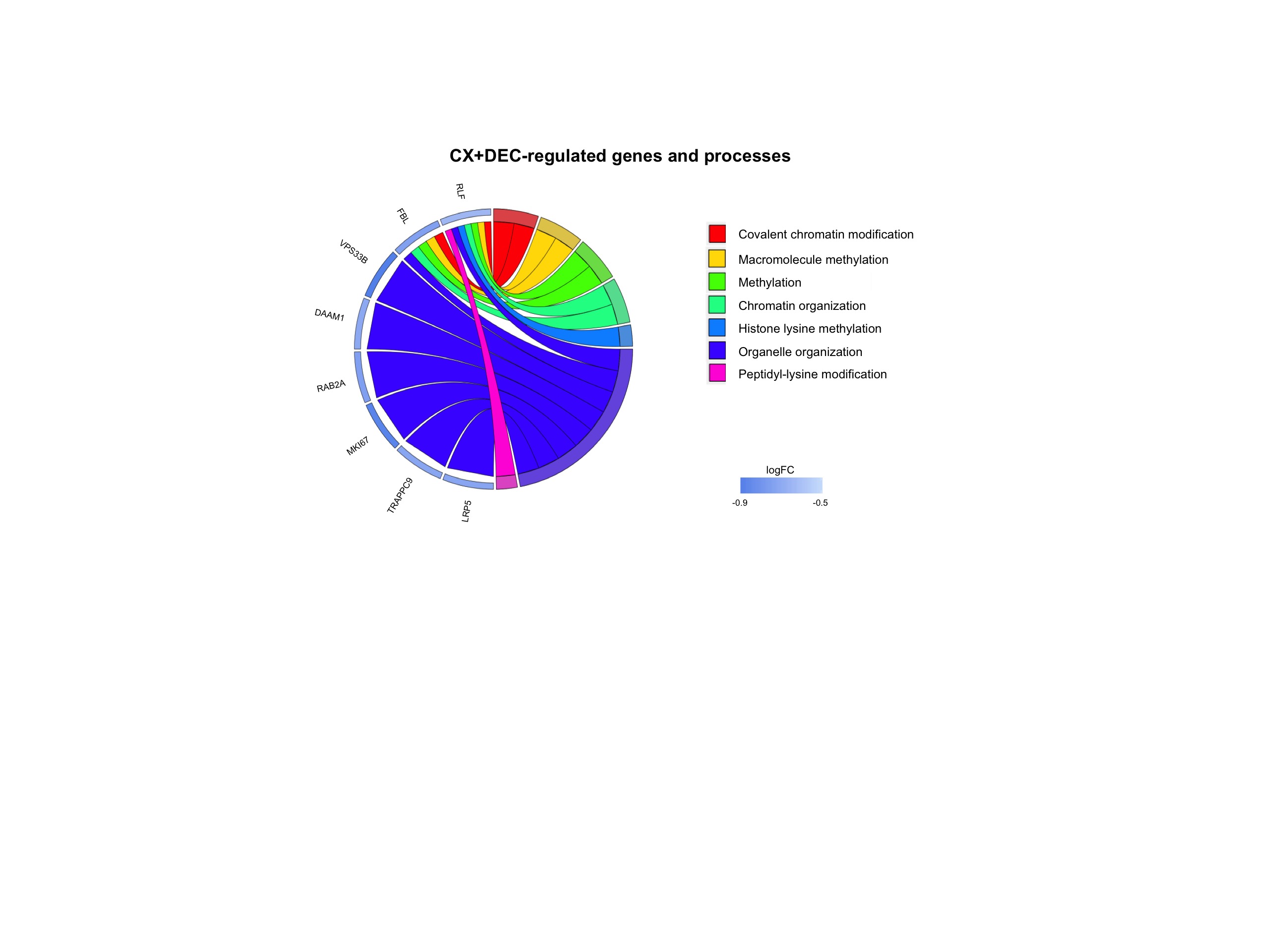

Supplement: Supplementary file 9 — Figure S2. Genes and processes highly regulated by combined CX-4945 and DEC incubation. Hypomethylated genes were assigned to Gene Ontology (GO) Terms. The chord plot shows the association between the top 7 enriched GO terms and top 30 hypomethylated genes. Genes are further classified by their fold change compared to control cells (blue rectangle). (JPG 41 kb) [file 12885_2019_5411_MOESM9_ESM.jpg]
