# Supplementary material for: Combined Casein Kinase II inhibition and epigenetic modulation in acute B-lymphoblastic leukemia
Source: BMC Cancer. 2019 Mar 6;19:202. doi: 10.1186/s12885-019-5411-0 (PMC6404304; doi:10.1186/s12885-019-5411-0)
Supplement: Supplementary file 11 — Table S9. Influence of CX and DEC on PTEN and CK2 promoter methylation beta values and respective fold changes (FC). (DOCX 14 kb) [file 12885_2019_5411_MOESM11_ESM.docx]

Supplemental Table 9: Influence of CX and DEC on PTEN and CK2 promoter methylation beta values and respective fold changes (FC)

| Target | DMSO | CX-4945 | DEC | CX+DEC | FC CX vs DMSO | DC DEC vs DMSO | FC CX+DEC vs DMSO |
| --- | --- | --- | --- | --- | --- | --- | --- |
| CSNK2A1 | 0,6050 | 0,6061 | 0,5437 | 0,5579 | 1,0067 | 0,9012 | 0,9102 |
| CSNK2A2 | 0,4995 | 0,4962 | 0,4502 | 0,4546 | 1,0039 | 0,8047 | 0,8389 |
| CSNK2B | 0,5981 | 0,5958 | 0,4813 | 0,5017 | 1,0082 | 0,8866 | 0,9167 |
| PTEN | 0,5847 | 0,5800 | 0,5184 | 0,5360 | 0,9785 | 0,9616 | 0,9741 |
